# DuBois-Reymond prize lecture 2024: Multi-neuron patch-clamp to uncover the neuronal and synaptic physiology in the human neocortex

**DOI:** 10.1007/s00424-026-03176-x

**Published:** 2026-07-11

**Authors:** Yangfan Peng, Franz Xaver Mittermaier

**Affiliations:** 1 Institute of Cell Biology and Neurobiology, Charité-Universitätsmedizin Berlin, Berlin, Germany; 2 Institute of Neurophysiology, Charité-Universitätsmedizin Berlin, Berlin, Germany; 3 Department of Neurology with Experimental Neurology, Charité- Universitätsmedizin Berlin, Berlin, Germany

**Keywords:** Patch-clamp, Human cortex, Multipatch, Synaptic connectivity, Pyramidal neuron subtypes, Human brain slices, Electrophysiology, Network motif

## Abstract

The physiology of neurons and synapses determines how networks in the brain process information. While these properties are well described in rodents, they are less well established for the human brain. Increasing availability of surgically resected human brain tissue, together with advances in electrophysiology, has enabled new insights into species-specific differences of the human cortical microcircuit. This review highlights recent progress in the physiology of layer 2 and 3 pyramidal neurons of the human temporal cortex, focusing on the multi-neuron patch-clamp approach. Methodological developments such as automated pipette cleaning now allow large-scale mapping of cellular electrophysiology and synaptic connectivity in human cortex. This approach reveals substantial functional heterogeneity among layer 2 and 3 pyramidal neurons with distinct intrinsic properties, morphology, and connectivity patterns. Complex network analyses further uncover human-specific properties, such as a directed network topology with random reciprocity and a decoupling of synaptic strength from connectivity. Network simulations suggest that these wiring patterns expand the computational capacity of cortical microcircuits. Together, these studies showcase the potential of multipatch recordings to bridge rodent and human neurophysiology and to establish an empirical basis for how cellular and synaptic diversity shapes human cortical microcircuits.

## Using surgically resected tissue to study human neurophysiology

Neuronal networks are determined by the intrinsic physiology of individual neurons and the synaptic function of the connections between them. Together, these features enable complex computations, such as higher cognitive functions of the human brain, which are attributed to the neocortex. Notably, the supragranular layer 2 and 3 (L2/3) of the human neocortex has undergone substantial evolutionary expansion, raising the question of whether its microcircuit organization provides the basis for specialized cortical computation [1]. While cross-species comparisons of neuronal and synaptic properties have been reviewed previously [2–4], we focus here on recent advances in human neuronal and synaptic physiology in L2/3 of the temporal cortex and introduce the multi-neuron patch-clamp approach which has uncovered novel principles of human microcircuit organization [5–10].

**Fig. 1:**
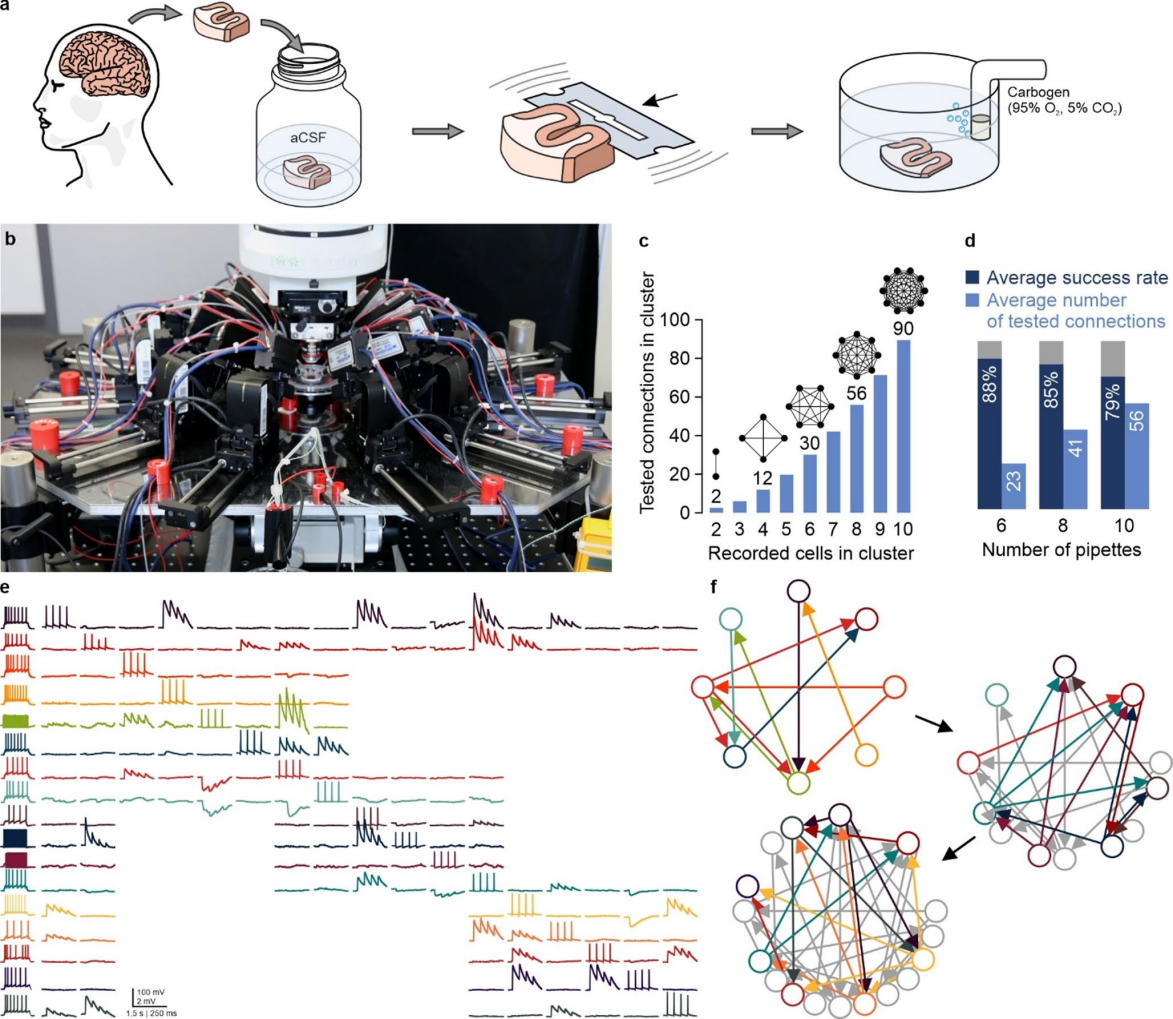
An optimized multipatch approach. (**a**) Schematic showing resection, transport, slice cutting and storage of surgically removed human brain tissue for acute brain slice experiments. aCSF: artificial cerebrospinal fluid. (**b**) Photograph of a 10-manipulator multipatch setup. (**c**) Bar graphs show number of testable connections for increasing number of simultaneously patched neurons. (**d**) Average success rate and tested connections were calculated based on size of recorded clusters in relation to number of pipettes on different setups. (**e**) Averaged voltage traces from seventeen neurons (rows, colors) recorded in one human slice. Rows correspond to presynaptic stimulation (four action potentials per neuron) and columns to postsynaptic responses. Eight neurons were recorded simultaneously in the first session, followed by two additional sessions after pipette cleaning and repatching of nearby neurons while some neurons were continuously recorded. (**f**) Graphs showing identified connections in each recording session. Overall, 38 connections were found among 150 tested connections. Adapted from Peng et al., 2019 [5], licensed under CC BY 4.0

An essential prerequisite for the growing field of human neuron physiology is the access and experimental use of fresh human brain tissue. During certain neurosurgical procedures for the treatment of brain tumors or epilepsy, neocortical tissue is resected along the surgical access corridor to reach the underlying lesion. This access tissue contains alive neurons as well as intact synaptic circuits and represents the closest available approximation to the healthy human brain. For electrophysiological experiments, the resected tissue is rapidly transferred from the operating theatre to the lab in ice-cold and oxygenated artificial cerebrospinal fluid for subsequent slicing and storing at room temperature (Fig. 1a). Brain slices kept under these conditions maintain neuronal viability and functional synaptic connectivity for more than 24 h [11–14], allowing investigation of human neuronal and synaptic function using electrophysiological methods.

The use of surgically resected human brain slices also has important limitations. Resected tissue samples are obtained from diverse patients that need brain surgery, introducing patient-specific factors as potential confounds. These factors could arise from underlying pathology, medication, age, sex or surgical procedure. As healthy control tissue is not available, studies have applied neuropathological assessment, correlation with disease factors, or used cross-disease controls (e.g. tumor vs. epilepsy) to assess potential impact or exclude the influence of these patient variables on the observed results [7, 15, 16]. Furthermore, additional factors during and after slice preparation are important to maintain slice viability and neuron physiology, such as slicing solution, slicing angle, and sterile conditions to reduce traumatic and inflammatory effects [11, 17]. To preserve slices for extended time periods, several studies have shown the feasibility of prolonged organotypic slice cultures [12, 13, 18].

Patch-clamp recording in resected human brain tissue has emerged as a key experimental approach to directly compare human neurons to established principles based on animal models, such as rodents. L2/3 pyramidal neurons are the most extensively studied neuronal population in humans and exhibit increased structural complexity and distinctive functional properties, characterized by elaborate dendritic arbors most evident in deeper L2/3 neurons [19–21]. Their apical dendrites exhibit active properties, including sodium-mediated spikes and a class of calcium-mediated action potentials not observed in rodents [22, 23]. In addition, human L2/3 pyramidal neurons show a higher density of hyperpolarization-activated cyclic nucleotide–gated (HCN) channels which is organized in a laminar depth–dependent manner with deeper neurons exhibiting more pronounced HCN-mediated properties [24, 25]. Furthermore, voltage-gated sodium and potassium channels at the soma of these neurons show kinetics distinct from those observed in rodents [26]. These functional specializations in human neurons (1) facilitate the efficient transfer of synaptic signals from distal dendrites to the soma [27], (2) enable them to encode fast synaptic input signals in their action potential output [28] and (3) ensure that the action potential waveform remains stable even during sustained firing [26, 29]. While these studies have uncovered unique properties of human pyramidal neurons, to what extent these features are ubiquitous or specific to a subpopulation of molecularly distinct neurons remains unknown.

In recent years, molecular techniques such as single-cell transcriptomics have advanced our understanding of the cellular diversity in the human brain [30, 31]. Gene expression profiling has enabled detailed comparisons of neocortical cell type composition across species [31, 32]. These studies indicate that broad neuronal classes are conserved between rodents and humans. Transcriptomic studies have also revealed species-specific differences, such as the relative abundance of cell types among humans, non-human primates, and mice [32, 33]. In addition, the degree of neuronal heterogeneity within conserved classes can vary across species. For example, L2/3 pyramidal neurons display greater molecular diversity in humans [16]. To link the molecular identity of a neuron to its functional properties, previous studies have combined whole-cell patch-clamp recordings with single-cell RNA sequencing (“Patch-seq”) [34]. This approach has shown that transcriptomically defined pyramidal neuron subtypes are associated with differences in intrinsic electrophysiological properties, but the correspondence is not complete and considerable variability remains across and within subtypes [16]. Thus, a comprehensive classification based on functional properties is yet lacking. Furthermore, the relationship between pyramidal neuron subtypes in L2/3 and the organization of synaptic circuitry is unknown and requires the simultaneous recording of cellular and synaptic properties.

While the electrophysiology, morphology and molecular properties of human neurons are well characterized, only few studies have addressed the physiological properties of human synaptic connections, which are usually defined by strength, kinetics, and plasticity [4]. For example, early studies found that single action potentials of pyramidal neurons can trigger complex di- and polysynaptic events in GABAergic interneurons via very large excitatory postsynaptic potentials (EPSP) [35–37]. Other groups have found that human synapses recover from short-term depression three to four times faster and that they can be much stronger and more reliable than rodent neocortical synapses [28, 38]. On the other hand, increased synaptic transmission by analog presynaptic potentials appears to be conserved across rodents and humans [39]. Thus, while previous human synaptic studies established unique human properties with respect to strength, kinetics, and plasticity, it remains unknown how these synaptic properties relate to cellular subtypes or to what extent they arise from interindividual differences or canonical principles of synaptic organization.

In the following sections, we focus on how the multi-neuron patch-clamp approach applied to human cortical tissue can help us resolve these open questions to uncover novel principles of human cellular and synaptic organization.

## The multi-neuron patch-clamp approach

The gold standard for functional characterization of unitary monosynaptic connections is performing paired whole-cell patch-clamp recording of two or more neurons in acute brain slices, which enables high–temporal-resolution measurements of excitatory and inhibitory synaptic currents and potentials [40]. Within the same experiment, both pre- and postsynaptic neurons can be electrophysiologically characterized and biocytin filling allows subsequent morphological reconstruction [41]. While recording a pair of neurons only samples two potential connections, one in each direction, increasing the number of simultaneously recorded neurons increases the sample size of tested connections [5]. For example, simultaneous patch-clamp recording of eight or ten neurons allows testing of up to 56 or 90 synaptic connections at once (Fig. 1b-d). Thus, the multi-neuron patch-clamp approach, also known as multipatch, is especially well suited to map connectivity and has been used to uncover cell-type and layer-specific connectivity across different brain regions in rodent brain slices [42–47].

Previous human multipatch studies have recorded pyramidal neurons across all layers of the human cortex and established the existence of recurrent connectivity with probabilities ranging from 12 to 16% [9], while showing differences in synaptic dynamics compared to mouse multipatch data [8]. On the other hand, studies focusing on human interneurons found conserved principles across species, such as depressing synapses onto parvalbumin-positive interneurons and facilitating synapses onto somatostatin-positive interneurons [48]. Beyond the cortex, other groups have also applied the multipatch approach to human hippocampal slices and found a much sparser connectivity (~ 1%) compared to the human neocortex [10]. In line with cortical synapses, synapses in the human hippocampus were also more reliable than those of rodents. Together, these rare and valuable human multipatch studies suggest that synaptic connectivity principles are only partially conserved across species, supporting a view that synaptic principles can exhibit human- and region-specific specializations.

While multipatch has been adopted by several groups, it remains a technically challenging method and comes with limitations. For example, penetration of brain slices with ten pipettes introduces mechanical stress on the slice affecting recording stability and potentially tissue integrity. Also, subsequent morphological reconstruction become more difficult with many simultaneously stained neurons with overlapping processes. One major bottleneck is the success rate of obtaining a stable whole-cell recording which depends on many factors, including slice quality, technical skill, and membrane properties. In our hands, we observed a drop in success rate from 88% to 79% when going from 6- to a 10-neuron multipatch setup (Fig. 1d). Because replacing pipettes during a multipatch experiment risks losing already patched neurons, an 80% success rate per neuron translates to only ~ 10% successful 10-neuron multipatch attempts.

To overcome this limitation, we developed an optimized multipatch approach incorporating automated pipette cleaning, which was described in a dedicated methods study that is briefly summarized here [5]. A critical component of this optimized approach is the patch pipette cleaning protocol described by Kolb et al. [49]. It involves dipping the pipette tip into a detergent solution with subsequent pressure-suction cycles to rinse the pipette tip and expel any residual detergent at the end. To implement this procedure in the multipatch approach, a custom recording chamber was constructed with an outer circular well for the cleaning solution. Automatization of the manipulator movements and the pipette pressure control system allowed pipette cleaning during ongoing experiments between individual patch attempts. By cleaning and reusing pipettes after a failed patch attempt, we were able to increase the number of neurons in one multipatch experiment, or cluster. On the 10-neuron multipatch setup, this “clean-to-complete” strategy increased the average cluster size from 7.9 to 9.2 with a success rate of 92%. The system could also be used to “clean-to-extend” the cluster by repatching new neurons with a subset of pipettes allowing probing of additional connections to already characterized neurons. We further demonstrated the feasibility of this approach in human brain slices by showing that a single 17-neuron multipatch cluster could identify 38 synaptic connections out of 150 tested (Fig. 1e-f). By operating two setups in parallel, recordings from up to 99 neurons and testing of up to 700 putative synaptic connections per human subject became feasible. Taken together, these methodological improvements laid the groundwork for subsequent large-scale studies of human neuronal and synaptic physiology.

Although the number of human multipatch studies remains limited, several groups have already established key principles of local synaptic connectivity in the human brain.

## Functional diversity of human pyramidal neurons and subtype-specific synaptic connectivity

**Fig. 2:**
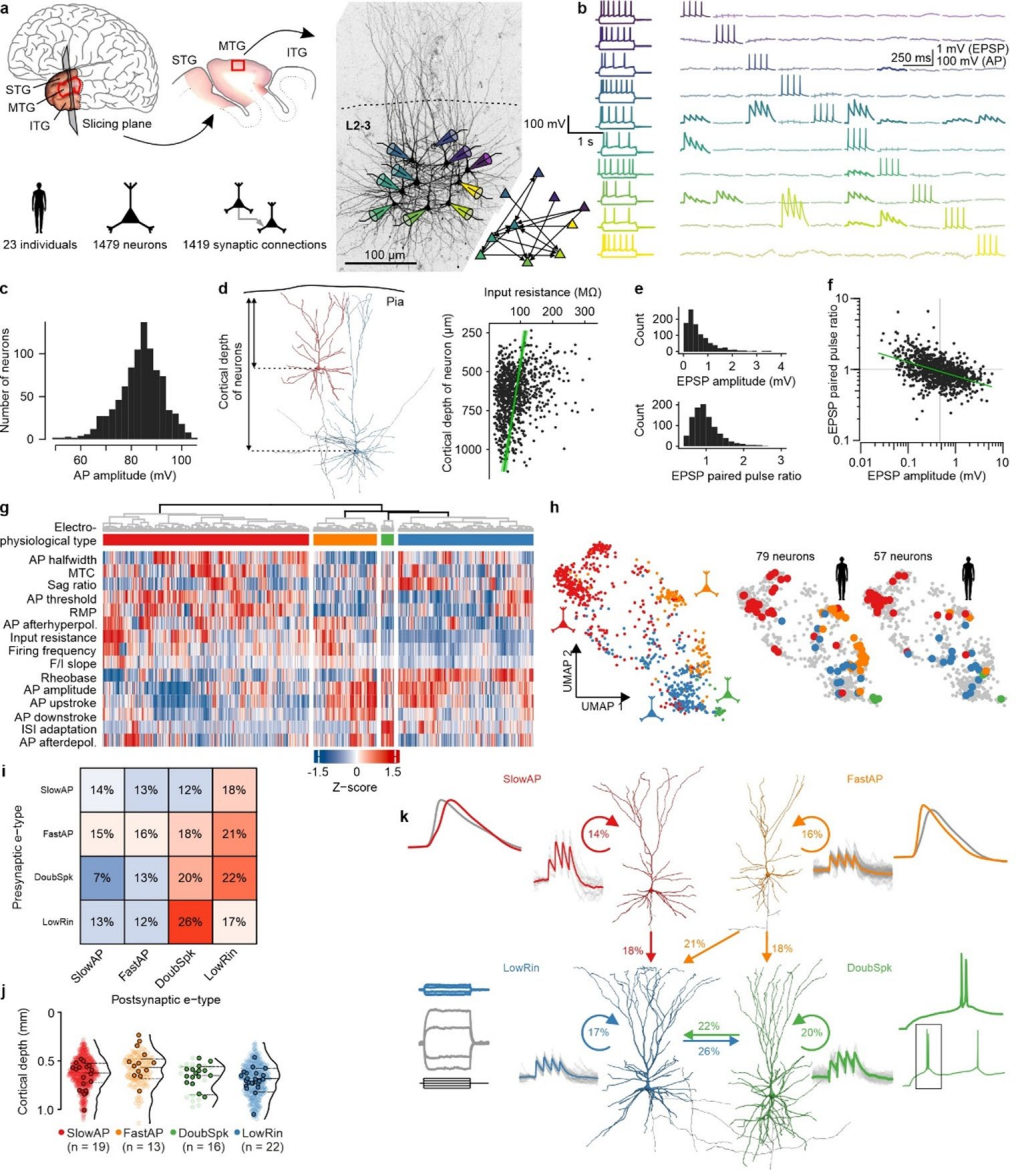
Large-scale multipatch analysis of human L2–3 pyramidal neurons reveals functional subtypes and their synaptic connectivity. (**a**) Experimental workflow for multipatch recordings from L2–3 pyramidal neurons. Acute brain slices were prepared from human middle temporal gyrus (MTG). Up to 10 neurons were recorded simultaneously using whole-cell patch-clamp, enabling identification of unitary synaptic connections between recorded cells. (**b**) Voltage traces of 10 L2/3 pyramidal neurons recorded in one brain slice. The first column shows voltage response of each neuron to 1 s long step currents used to obtain intrinsic electrophysiological properties. Connectivity matrix showing presynaptic stimulation (rows; four action potentials per neuron) and corresponding postsynaptic responses (columns). (**c**) Histogram of action potential (AP) amplitudes across all neurons in the dataset. (**d**) Left, two neurons located at different cortical depths. Right, relationship between cortical depth and input resistance. (**e**) Histograms of excitatory postsynaptic potential (EPSP) amplitude and paired-pulse ratio for all unitary synaptic connections. (**f**) Power-law relationship between EPSP amplitude and paired-pulse ratio. (**g**) Clustergram of hierarchical clustering analysis identifying four electrophysiological types (e-types). Rows represent the 15 intrinsic electrophysiological parameters; columns represent individual neurons. Colors indicate z-scored parameter values. The dendrogram is shown above (black lines denote statistically significant clusters). (**h**) UMAP projection of the 15-dimensional electrophysiological parameter space. Colors indicate e-types. (**i**) Matrix of connection probabilities for all 16 combinations of pre- and postsynaptic e-types. (**j**) Distribution shows cortical depth of different recorded e-types. Circled dots indicate locations of reconstructed neurons. (**k**) Summary schematic of circuit architecture and representative morphological reconstruction by electrophysiological subtype. Adapted from Planert et al., 2025 [6], licensed under CC BY 4.0

Human L2/3 pyramidal neurons exhibit substantial morphological and molecular heterogeneity [16, 20, 21, 31]. However, it was previously unclear to what extent this molecular diversity relates to their functional properties, and how cellular heterogeneity determines synaptic connectivity within the cortical microcircuit. Using our multipatch approach, we recorded a large-scale dataset consisting of more than 1,400 pyramidal neurons and identified over 1,400 monosynaptic excitatory connections (Fig. 2a-b) in L2/3 of the human temporal cortex. In the following sections, we will highlight the key findings from this study [6]. We found a large heterogeneity among L2/3 pyramidal neurons at the level of intrinsic electrophysiological properties (resting membrane potential, AP shape, rheobase, etc.; Fig. 2c). Some parameters, including the input-resistance and sag-ratio, varied systematically with cortical depth of neurons (i.e., the distance of the soma from the pial surface; Fig. 2d), in line with previous studies [24, 25]. However, depth explained only a small fraction of the total functional variance, suggesting additional sources of variability.

To test whether the observed diversity could reflect distinct functional subtypes, we used unsupervised clustering of intrinsic electrophysiological parameters to identify four subtypes (e-types; Fig. 2g-h). E-types did not show a simple correspondence with previously described transcriptomic subtypes [16, 31], underscoring that physiology-based classifications capture complementary aspects of neuronal diversity. Each e-type displayed characteristic intrinsic properties (Fig. 2g). ‘SlowAP’ neurons were highly excitable but generated action potentials with slower kinetics. ‘FastAP’ neurons exhibited rapid action potential kinetics and more hyperpolarized resting membrane potentials. ‘LowRin’ neurons were less excitable, with low input resistance and higher rheobase, while the rare ‘DoubSpk’ subtype showed characteristic initial doublet firing. Electrophysiological diversity was further paralleled by morphological specialization (Fig. 2j). Three-dimensional reconstructions revealed systematic differences in soma size, dendritic length, and branching patterns across e-types. For example, LowRin neurons had more elaborate apical dendrites and larger somata, consistent with their low input resistance and high rheobase. FastAP neurons had more compact dendritic arbors. DoubSpk neurons had long and complex basal dendrites. These morpho-electric differences imply that e-types process synaptic input in distinct ways and, as a result, contribute differently to overall network activity.

At the synaptic level, the overall connection probability among L2/3 pyramidal neurons was 15.8%. Synaptic amplitudes ranged from 0.1 to 6 mV, varying by orders of magnitude and short-term dynamics spanned a wide range from paired-pulse facilitation to depression. These distributions followed a heavy-tailed distribution, with many weak facilitating and few strong depressing synapses (Fig. 2e-f). We further found that synaptic properties were dependent on pyramidal neuron subtypes. LowRin neurons received disproportionately more excitatory inputs, whereas SlowAP neurons received less than expected (Fig. 2i). This asymmetry suggests a circuit architecture in which LowRin neurons act as integrative hubs within L2/3. Synaptic function was also subtype-specific. Homotypic connections were particularly distinctive: SlowAP-to-SlowAP synapses were among the strongest and most depressing, while FastAP-to-FastAP synapses were weaker and displayed paired-pulse facilitation. Thus, e-type identity does not only reflect intrinsic properties but also determines how information is integrated and propagated by a neuron. Finally, both the broad distribution of synaptic properties and their dependence on e-types were conserved across individual subjects. For instance, in 10 out of 11 subjects, the homotypic SlowAP-to-SlowAP synapses were stronger compared to the homotypic FastAP-to-FastAP synapses. This consistency across individuals supports the conclusion that the observed microcircuit rules reflect general principles of human cortical L2/3.

Taken together, this study demonstrated that functional diversity among human L2/3 pyramidal neurons can be captured by e-types with specific morphology, connectivity, and synaptic dynamics. We propose that this fine-grained organization gives rise to specialized excitatory subnetworks within L2/3 of the cortex, enabling differential integration and transmission of information.

## Higher-order network structure in the human cortex

While the previous study established that human synaptic connections depend on the cellular properties of the pre- and postsynaptic neurons, larger sets of simultaneously recorded neurons (multipatch cluster) further enable the investigation of higher-order network structure which we addressed in a separate study [7]. Because even multipatch recordings sample only a small fraction of the underlying network, its global structure is commonly inferred by analyzing subnetwork motifs, defined as non-isomorphic configurations of typically three or more nodes [50]. Motif frequencies observed in the data are then compared to those obtained from random network models to identify over- or underrepresented connectivity patterns. Here, the null hypothesis is typically an Erdős–Rényi random graph, in which individual connections are established independently with a probability defined by the overall first-order connection probability. Importantly, many rodent multipatch studies have consistently reported non-random connectivity patterns among pyramidal neurons with the rate of reciprocal connections increasing up to 4-fold and their synaptic amplitudes being higher than those of unidirectional connections [42, 51]. This suggests a network architecture that supports feature amplification through strong, reciprocal interactions between similarly tuned neurons [52]. However, whether these principles extend to the human cortex was not established.

**Fig. 3:**
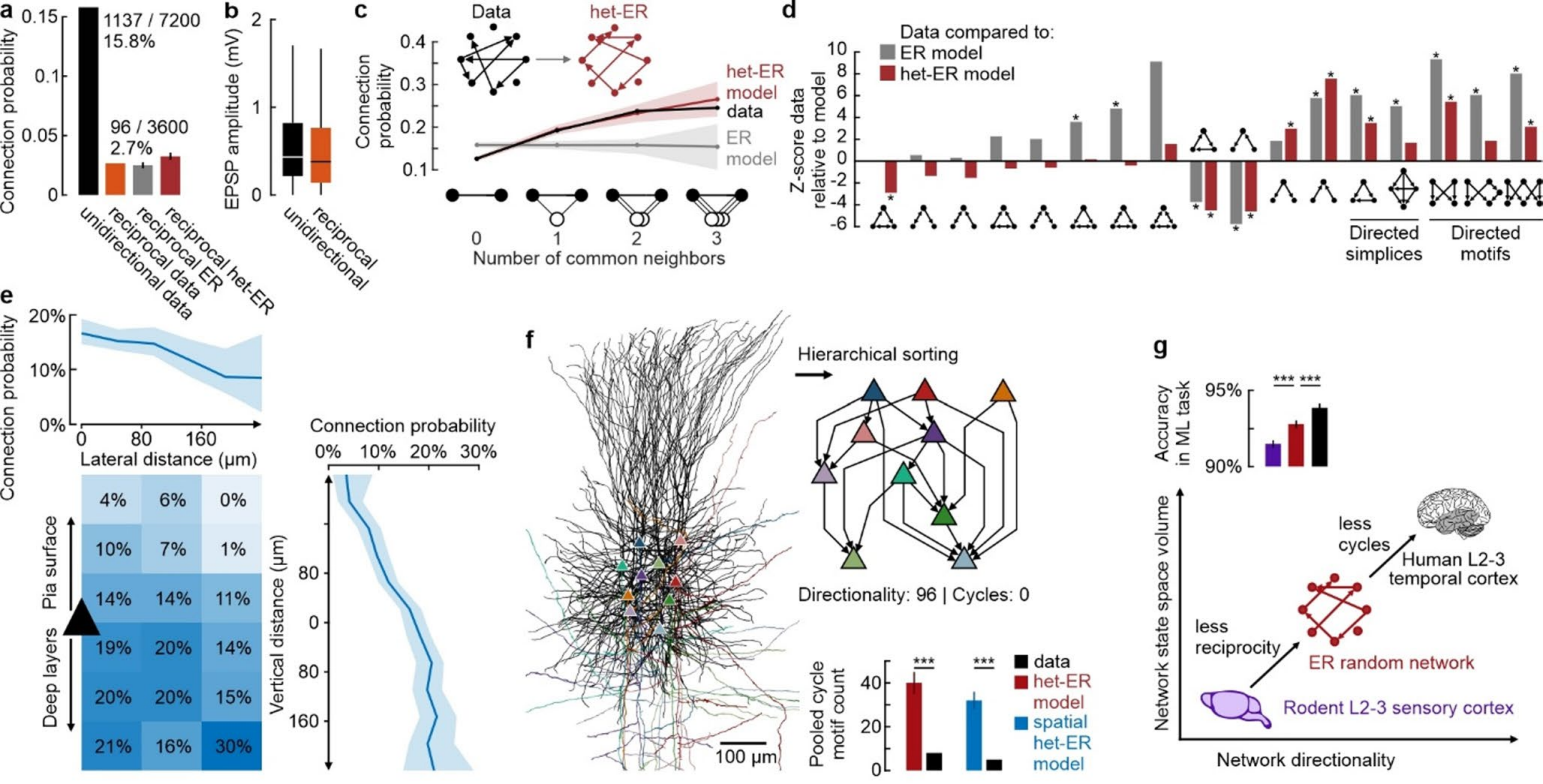
Higher-order network motifs and directed connectivity. (**a**) Probabilities of unidirectional (black) and reciprocal (orange) connections. Grey and red represent expected reciprocity based on random models. Error bars indicate SD in models. (**b**) Box plots show EPSP amplitudes compared between unidirectional and reciprocal pairs with no significant difference. (**c**) Connection probability (lines) of pairs with different number of common neighbors (open circles) in data (black) and in random models (grey, red). (**d**) Normalized over- or underrepresentation of different network motifs in the data compared to random models (grey, red). Asterisks mark statistically significant deviations from the random model. (**e**) Connection probability for 80 × 80 μm bins around the presynaptic soma. Line plots show increasing connectivity towards deeper neurons and a lateral drop in connectivity. (**f**) Reconstruction of the cluster with highest directionality score. Colored triangles represent soma locations, axons colored for each neuron, all dendrites in black. Graph shows hierarchically sorted connectivity of the cluster. Bar graphs show total motif count of triadic cyclic motifs in the data (black bars) compared to the median count in the random model simulations (colored bars). (**g**) Schematic representation of the relationship between the directionality and state space volume of the network. Bar graph shows the performance in a speech recognition task of simulated networks that are constrained by different connectivity principles. Adapted from Peng et al., 2024 [7] with permission from AAAS

In our higher-order connectivity study [7], we focused on complex network patterns to identify conserved and divergent principles of network organization. We found that the rate of reciprocal connections in the human dataset was comparable to that predicted by random network models that assume synaptic connections to be established independently at the overall rate of 15.8% (Fig. 3a). In addition, reciprocal connections did not exhibit larger EPSP amplitudes than unidirectional connections and showed no significant relationship to the local connection probability in the recording cluster (Fig. 3b). These results diverge from previous assumptions based on rodent studies [42, 51]. While species-specific specializations are one potential explanation, methodological differences are also likely to contribute, including variations in brain region, age range, and sampling strategies such as cluster size and intersomatic distance [53]. We further observed that connection probability within individual recording clusters varied widely from 0 to 50%, even within the same tissue sample. This pronounced cluster-to-cluster variability (coefficient of variation ~ 0.5) cannot be explained by slice cutting effects and is consistent with a highly skewed distribution of connections across individual neurons. Incorporating this variability into a “heterogenous Erdős-Rényi model” (het-ER) was sufficient to fully account for the increased connectivity observed between neuron pairs with many common neighbors (Fig. 3c). Thus, the so-called “common-neighbor rule”, previously described as a key wiring principle in rodent cortex [42], could be understood as an emergent consequence of the non-random connectivity at the single neuron level. Extending this analysis to triadic motifs consisting of three neurons, we found that the het-ER model also accounted for many non-random motif occurrences (Fig. 3d). However, directed motifs of three or more neurons remained significantly overrepresented, suggesting an increased directionality of the network that goes beyond the variability of connection probability across recording clusters.

Because spatial location is a well-established constraint on synaptic connectivity, we next examined how connection probability depends on the relative spatial position of the neuron pair. We found that connectivity along the vertical axis was strongly asymmetric. While connection probability decreased toward apical locations, it increased toward deeper cortical locations along the vertical dimension (Fig. 3e). While increased connectivity towards basal directions has been observed in rodents [42], our analysis extends this principle to the human cortex and further demonstrates that this bias persists over vertical distances exceeding 200 μm and that it can be observed across all sampled laminar depths across L2/3. One important consideration is the potential influence of dendritic filtering, which could contribute to an underestimation of weak synaptic inputs at distal dendrites. For example, in human L5 pyramidal neurons, apical dendritic signals were attenuated by 50% at distances of around 450 μm, similar to those observed in rats [54]. While pyramidal neurons in the rodent hippocampus exhibit multiple mechanisms to compensate for such filtering [55], some of these mechanisms appeared less prominent in human L5 pyramidal neurons [54]. However, given that L2/3 pyramidal neurons have specialized active dendritic properties [23], and that our analysis was restricted to relatively short intersomatic distances, within which EPSP amplitude did not correlate with vertical distance, dendritic filtering is unlikely to be a dominant factor underlying the observed vertical asymmetry in connectivity.

To determine whether this asymmetry could explain the increased directionality, we incorporated the spatial connectivity into the het-ER model to generate a spatial het-ER model. While this model reduced the overrepresentation of directed motifs by > 50%, a substantial residual directionality remained unexplained, suggesting additional feed-forward structure in the network independent of spatial connectivity. We thus analysed cyclic motifs. By leveraging larger multipatch recording clusters, we quantified cyclic motifs with up to six neurons and found a consistent underrepresentation of such patterns. This meant that even densely connected clusters could be rearranged into fully directed acyclic graphs (Fig. 3f). This apparent lack of cycles revealed a previously unrecognized aspect of network organization.

Taken together, while skewed and directed connectivity reflect conserved principles of cortical network organization, our study identified novel principles of connectivity in the human L2/3 temporal cortex. Specifically, we found a directed and acyclic network topology with a decoupling between synaptic connectivity and synaptic strength. Such a specific network architecture shapes information flow and computation in human cortical networks, which we further explored through network simulations.

## Computational capacity of the human cortical microcircuit

To understand how each of the identified network principles could impact the computational capacity of the human cortical microcircuit, we simulated empirically constrained recurrent neural network models [7]. The model comprised excitatory (E) and a simplified fast-spiking-like inhibitory (I) population, with connectivity constrained by the empirical data (15% E-E, 25% E-I, and 25% I-E). Synaptic weights were sampled from uniform distributions and rescaled to maintain a balanced excitation-inhibition regime. To ensure stable dynamics, the largest real part of the eigenvalue spectrum was normalized to 1, placing the network near criticality and enabling fair comparisons across different network topologies. We then varied different network features identified in our study, namely network directionality, reciprocity and correlation between connectivity and synaptic weights. Using random matrix theory [56], we focused on the effect of these properties on the “neural state space volume” which reflects how many distinct activity states the neural network can occupy. We found that all features identified in the human cortex, namely random reciprocity, acyclicity and independence of weights from connectivity, increased the state space volume compared to rodent-like network models. These relative effects were robust in simulations with lower connectivity or lower synaptic weights. However, this modelling approach has limitations as it was intentionally simplified. It reduced the cortical microcircuit to a rate-based E/I network and does not capture the functional cellular diversity and subtype-specific connectivity.

To explore the functional implication of this finding, we trained these networks on a machine learning spoken digit recognition task to model the language processing within the temporal lobe. We found that all network features increased the task performance. Specifically, compared to a random network with 200 neurons, a rodent-like network with higher reciprocity would need twice as many neurons, whereas an acyclic human-like network would need 25% less neurons to achieve equal performance (Fig. 3g). Taken together, our simulation results suggest that the identified connectivity principles in the human temporal cortex L2/3 could make it more energy-efficient and support more complex computations, such as speech processing, a hallmark feature of the temporal lobe [57].

The computational capacity of a network is not only constrained by its connectivity structure, but also by the functional properties of the individual neurons and their synaptic interactions. Cortical neurons are highly diverse with the most fundamental classification of glutamatergic excitatory and GABAergic inhibitory neurons. Previous studies have reported increased subtype diversity in both neuronal classes [16, 58]. Our dataset on human pyramidal neurons provides key empirical evidence for extensive functional heterogeneity within and across neuronal subtypes at both the cellular and synaptic levels [6, 7]. Importantly, such variability can only be partially explained by subtype classification and is frequently observed within cell types [59, 60]. Such within-type heterogeneity has been highlighted as an important constraint with rich computational properties [61, 62]. Together, our findings support the idea that the increased subtype diversity and substantial within-type heterogeneity in human pyramidal neurons may increase the computational capacity of cortical microcircuits.

## Outlook

In this review, we have highlighted how emerging technologies, together with valuable access to resected human brain tissue, have enabled a renewed and rapidly growing effort to characterize the human cortex at the cellular and synaptic level. While recent studies revealed both shared and divergent principles compared to rodent cortex, many important questions remain open:

1. To what extent can functionally defined subtypes be mapped onto transcriptomic subtypes?
2. Do non-random connectivity principles observed in L2/3 extend to deeper cortical layers and its subtype diversity?
3. Does the inhibitory microcircuit exhibit similarly structured connectivity, and how is it dependent on neuronal subtypes?
4. How do patient-specific factors shape cellular and synaptic diversity?

Finally, addressing these questions is important not only for defining species-specific differences, but also for providing the mechanistic foundation needed to interpret single-neuron recordings from human patients, which are becoming increasingly feasible through rapid technological advances [63]. A deeper understanding of human neuronal processing will be crucial for developing translational applications that target cellular and circuit-level mechanisms underlying brain disease.

## Data Availability

No datasets were generated or analysed during the current study.
